# The Protective Effect of Aspirin Eugenol Ester on Paraquat-Induced Acute Liver Injury Rats

**DOI:** 10.3389/fmed.2020.589011

**Published:** 2020-12-17

**Authors:** Zhen-Dong Zhang, Ya-Jun Yang, Xi-Wang Liu, Zhe Qin, Shi-Hong Li, Jian-Yong Li

**Affiliations:** Key Lab of New Animal Drug Project of Gansu Province, Key Lab of Veterinary Pharmaceutical Development of Ministry of Agriculture and Rural Affairs, Lanzhou Institute of Husbandry and Pharmaceutical Sciences of CAAS, Lanzhou, China

**Keywords:** aspirin eugnol ester, paraquat, metabolites, hepatotoxicity, antioxidation

## Abstract

Aspirin eugenol ester (AEE) possesses anti-inflammatory and anti-oxidative effects. The study was conducted to evaluate the protective effect of AEE on paraquat-induced acute liver injury (ALI) in rats. AEE was against ALI by decreasing alanine transaminase and aspartate transaminase levels in blood, increasing superoxide dismutase, catalase, and glutathione peroxidase levels, and decreasing malondialdehyde levels in blood and liver. A total of 32 metabolites were identified as biomarkers by using metabolite analysis of liver homogenate based on ultra-performance liquid chromatography-tandem mass spectrometry, which belonged to purine metabolism, phenylalanine, tyrosine and tryptophan biosynthesis, glycerophospholipid metabolism, primary bile acid biosynthesis, aminoacyl-tRNA biosynthesis, phenylalanine metabolism, histidine metabolism, pantothenate, and CoA biosynthesis, ether lipid metabolism, beta-Alanine metabolism, lysine degradation, cysteine, and methionine metabolism. Western blotting analyses showed that Bax, cytochrome C, caspase-3, caspase-9, and apoptosis-inducing factor expression levels were obviously decreased, whereas Bcl-2 expression levels obviously increased after AEE treatment. AEE exhibited protective effects on PQ-induced ALI, and the underlying mechanism is correlated with antioxidants that regulate amino acid, phospholipid and energy metabolism metabolic pathway disorders and alleviate liver mitochondria apoptosis.

## Introduction

PQ is a non-selective herbicide with excellent effect, which has been widely used in the world for many years (1–3). PQ is extremely toxic to humans (4, 5). Studies have shown that when taking about 10 ml PQ, patients can die of multiple organ failure a few hours later (6). The accumulation of PQ can damage the main organs such as lung, kidney, liver and heart (7). It is reported that the liver is one of the main target organs of PQ poisoning, which is often accompanied by the formation of free radicals (8, 9). The liver is the main metabolic and detoxifying organ of the human body (10, 11). A multiple potentially harmful stimuli challenge the liver, including free radicals. It is well known that drugs and other substances are further transformed and metabolized after being absorbed by the body, resulting in the production of free radicals in the liver. Excessive free radicals produce oxidative stress on the liver, which in turn leads to oxidative damage to the liver (12).

Currently, the molecular mechanism of hepatotoxicity induced by PQ is not completely understood. It is known that the redox response is one of the main factors involved in the toxic effects of PQ (13). It has been reported that PQ molecules can interfere with the electron transport chain and then inhibit the synthesis of NADPH (14). Excessive production of ROS was observed during PQ poisoning, indicating that oxidative stress was involved in the pathological changes induced by PQ. Excessive ROS and excessive free radicals lead to oxidative stress by destroying DNA, proteins and lipids (15). Therefore, the premise of the toxic effect of PQ is its induced oxidative stress. At present, the main methods for the treatment of PQ poisoning are immunosuppressant and hemodialysis (16). Existing clinical treatments for severe PQ poisoning only relieve symptoms (17). In recent decades, new drugs to treat the toxicity of PQ have been developed. In the early stages of poisoning, the use of antioxidants has been shown to effectively reduce the damage of PQ to organs. Therefore, it is imperative to develop potential effective drugs for the treatment of PQ poisoning.

AEE is a new potential pharmaceutical compound possessing anti-inflammatory and anti-oxidative stress pharmacological activity (18–22). The effect of AEE against H_2_O_2_-induced oxidative stress of human umbilical vein endothelial cells is consistent with the AEE-enhanced expression of Bcl-2 and Nrf2 (18, 23). It has been well documented that AEE could alleviate H_2_O_2_-induced dysfunction of mitochondria, the generation of ROS productions and the increase of apoptosis via enhancing the expression of Bcl-2 and Nrf2 (18, 23). It is well known that the dysfunction of mitochondria could release cytochrome C, apoptosis inducing factor (AIF), and other factor into cytoplasm to mediate downstream apoptotic signals causing cell apoptosis (24, 25), while the exacerbation of reactive oxygen species (ROS) induced by the dysfunction of mitochondria is also vital incentive of cell apoptosis (26, 27).

## Materials and Methods

### Chemicals

AEE (99.5%) was prepared in Lanzhou Institute of Husbandry and Pharmaceutical Sciences of CAAS (Lanzhou, China). MS-gradeacetonitrile was purchased from Thermo Fisher Scientific (Waltham, MA, USA). Formic Acid (98.0%, for LC-MS) was purchased from Tokyo Chemical Industry (Shanghai, China). Catalase assay kit was purchased from Solarbio (Beijing, China). Glutathione peroxidase (GPx), GSH and GSSG assay kit, superoxide dismutase (SOD), and malondialdehyde (MDA) assay kit were purchased from Beyotime (Shanghai, China). Caspase-3 assay kit was purchased from Jianglai Chemical Biotechnology (Shanghai, China). The antibodies of Caspase-9, Caspase-3, Bax, Bcl-2, Cyt C, AIF, and IgG were purchased from abcam (Shanghai, China). Alanine aminotransferase kit and aspartate aminotransferase kit were purchased from Mlbio (Shanghai, China).

### Animal Experiment

Eighteen male specific pathogen-free SD rats (6 weeks old) weighing 120–130 g were purchased from the Laboratory Animal Center of Lanzhou Veterinary Research Institute (Lanzhou, China). All animals were placed in groups in SPF-class housing of laboratory at a controlled relative humidity (55–65%), 12 h light/dark cycle and temperature (24 ± 2°C). The rats were randomly divided into three groups (*n* = 6): (1) control group, in which rats were administrated equivalent saline by intraperitoneal injection (ip); (2) PQ group, in which rats were administrated PQ (20 mg/kg body weight, ip) (28–30); (3) AEE groups, in which rats were pre-administrated AEE (54 mg/kg/day body weight) by gavage once a day for 1 week before being administrated PQ. The rats in the different groups were sacrificed after a single intraperitoneal injection of 20 mg/kg PQ for 24 h. All experimental protocols and procedures were approved by the Institutional Animal Care and Use Committee of Lanzhou Institute of Husbandry and Pharmaceutical Science of Chinese Academy of Agricultural Sciences (Approval No. NKMYD201907018; Approval Date: 18 July 2019). Animal welfare and experimental procedures were performed strictly in accordance with the Guidelines for the Care and Use of Laboratory Animals issued by the US National Institutes of Health.

### Metabonomic Analysis

#### Hepatic Tissue Sample Preparation

The hepatic tissue samples were homogenized with ice-cold physiological saline (10%, wt%, 1 g tissue in 10 mL of physiological saline) in an Ultra Turrax tissue homogenizer. After vortex mixing for 3 min, the samples were centrifuged at 12,000 rpm for 10 min at 4°C. The supernatant was subsequently analyzed by UPLC-QTOF-MS/MS.

#### UPLC-QTOF-MS/MS Conditions

Liquid chromatography was executed on DAD 1290 UPLC system (Agilent Technologies Inc., California, USA). Separation was performed on an Agilent SB C18 RRHD Column (2.1 × 150 mm, 1.8 μm). The temperature of the column was set to 35°C. Injection volume was 3 μL and autosampler temperature was set at 4°C. Mobile phase A consisted of water containing 0.1% formic acid and mobile phase B was acetonitrile containing 0.1% formic acid at a flow rate of 0.3 mL/min. The gradient elution of A was as follows: 98%A from 0 to 2 min, 98–55% A from 2 to 9 min, 55–30% A from 9 to 15 min, 30–2% A from 15 to 22 min, 2% A from 22 to 23 min, 2–98% A from 23 to 24 min and held at 98% A from 24 to 27 min. The mass spectrometer was operated in both positive and negative ionization modes. The fragment voltage was set to 135V and the skimmer voltage was set to 65 V. In positive ion mode, capillary voltage was 4.0 KV, while in negative ion mode, it was 3.5 KV. The temperature and the flow of the drying gas were 350°C and 10 L/min, respectively. The nebulizer pressure was set to 45 psig. Ions were scanned over a region of 50–1000 m/z.

#### Metabolomics Data Analysis

The raw MS data were initially processed with the Mass Profiler Professional (MPP) software (Agilent Technologies, USA) to filter noise, correct the baseline, align peaks, and identity and quantify peaks. The match tolerance of mass span is 10 ppm, and the match tolerance of retention time's span is 0.10 min. The obtained data were imported into SIMCA-P (version 13.0, Umetrics AB, Umea, Sweden), where a principal component analysis (PCA) and partial least squares discriminant analysis (OPLS-DA) were performed on the dataset. The quality of OPLS-DA models was described by R^2^X, R^2^Y, and Q^2^, and its validity was evaluated by performing permutation testing (with 200 permutations). The variable importance in the projection (VIP > 1) value of the validated OPLS-DA model and the *p* values from one-way ANOVA (*p* < 0.05) were used as the measurement indices to select potential metabolites. Metabolites were identified through a mass-based search followed by manual verification. Accurate mass values of the molecular ions of interest in TOF-MS data were searched against METLIN and Human Metabolome Database (HMDB). Then, an MS/MS analysis was conducted to confirm the structure of potential biomarkers by matching the masses of the fragments. The parent ion mass tolerance is ±10 ppm and mass/charge (m/z) of products tolerance is ±10 ppm. The clustering analysis of the potential biomarkers and pathway analysis were performed using MetaboAnalyst 4.0 and the metabolic pathways were identified using the KEGG database.

### Histopathology

Liver specimens were fixed with 10% formaldehyde. After fixation, the liver tissue was embedded in paraffin wax, sectioned to a thickness of 5 μm and stained with hematoxylin-eosin staining.

### Analysis of MDA, SOD, Caspase-3, GSH/GSSH, and GPx

The levels of MDA, SOD and the activity of caspase-3, the ratio of GSH/GSSH and GPx in the rat serum were assessed using the corresponding commercial kits according to the manufacturer's protocols.

### Protein Expression Analysis

The expression of AIF, Bax, Bcl-2, Caspase-3, Caspase-9, and Cyt c among different treatment was assessed by Western blot analysis. In brief, total protein of the liver was extracted using RIPA, quantified by bicinchoninic acid (BCA) method, and separated by precast SDS-PAGE Gel (15%, 4–20%). The separated proteins were transferred onto polyvinylidene fluoride (PVDF) membrane using standard procedures. Blots were incubated with the primary antibody followed by horseradish peroxidase-conjugated secondary antibody. Results were detected using the G: Box Chemi XRQ Imaging System (Cambridge, UK).

## Results

### AEE Reduces PQ-Induced Liver Injury in Rat

**Figure 1:**
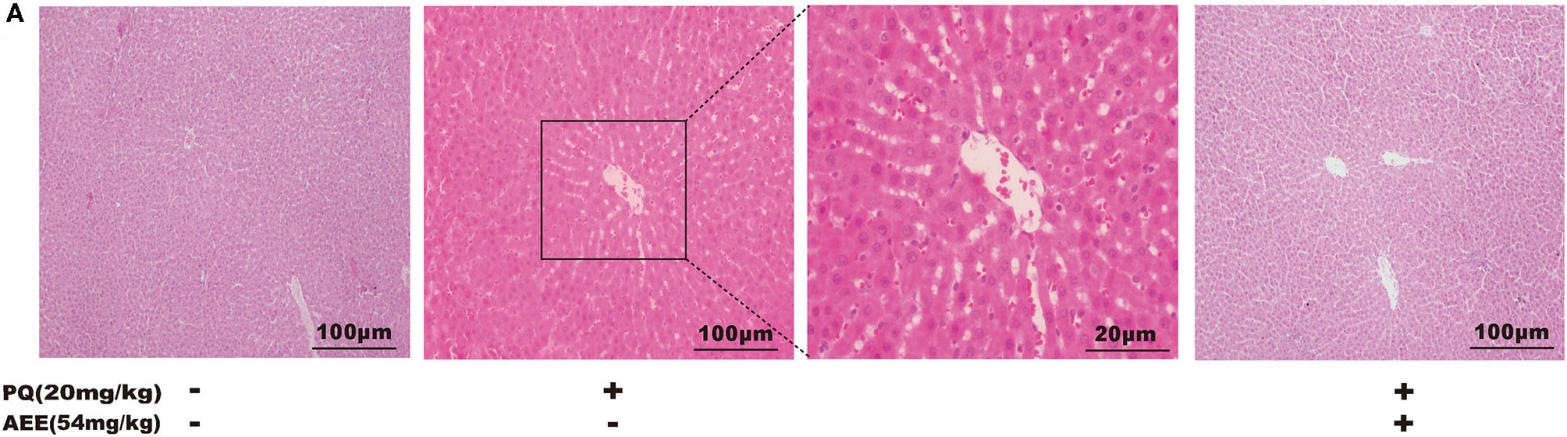
AEE reduces PQ-induced liver injury in rat. **(A)** Histopathological H&E staining of rat liver tissue (scale bar = 100 μm). Values are presented as the means ± SD where applicable (*n* = 6).

To verify whether AEE has a protective effect on PQ-induced hepatotoxicity *in vivo*, we explored the effect of AEE pretreatment on PQ-induced liver injury in rats. The results showed that PQ (20 mg/kg) could significantly cause liver tissue necrosis, cell atrophy and portal hyperemia in rats. Pretreatment with 54 mg/kg AEE for seven consecutive days by gavage markedly attenuated the pathological injury of liver tissue induced by PQ (Figure 1). The results showed that AEE could effectively reduce the liver injury induced by PQ in rats.

### AEE Attenuates PQ-Induced Oxidative Stress in the Liver of Rats

**Figure 2:**
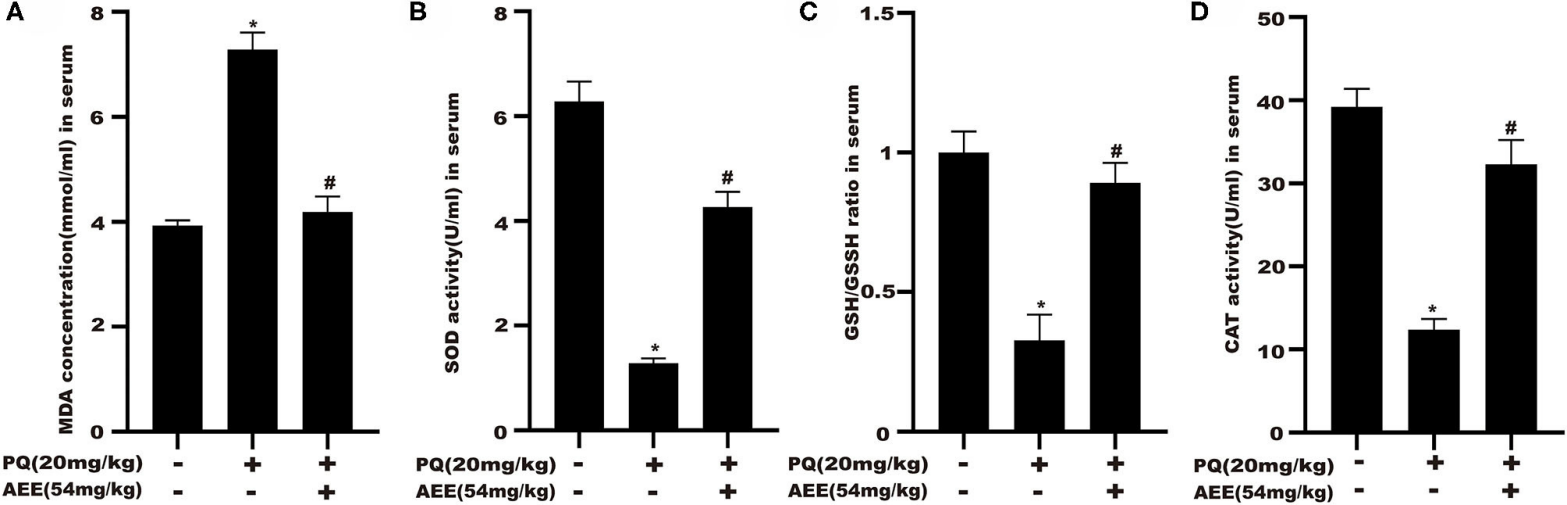
AEE attenuates PQ-induced oxidative stress in the liver of rat. **(A)** The activity of malondialdehyde (MDA) in serum of different treatment groups was detected. **(B)** The activity of superoxide dismutase (SOD) in serum of different treatment groups was detected. **(C)** The ratio of reduced glutathione/oxidized glutathione disulfide (GSH/GSSH) in serum different treatment groups was detected. **(D)** The activity of catalase (CAT) in serum of different treatment groups was detected. Values are presented as the means ± SD where applicable (*n* = 6). *p < 0.05 compared with the control group; ^#^*p* < 0.05 compared with the PQ group.

The results for CAT, MDA, SOD, GPx, and GSH/GSSH ratio in serum were shown in Figure 2. AEE significantly attenuated the increase in MDA and prevented the decrease in CAT, SOD, GPx activity, GSH/GSSH ratio caused by PQ in rats (Figure 2). These results suggested that AEE could effectively inhibit oxidative stress induced by PQ in rat liver.

### Metabolomics Analysis of AEE Effect on PQ-Induced ALI in Rats

#### Analysis of Liver Metabolites

**Figure 3:**
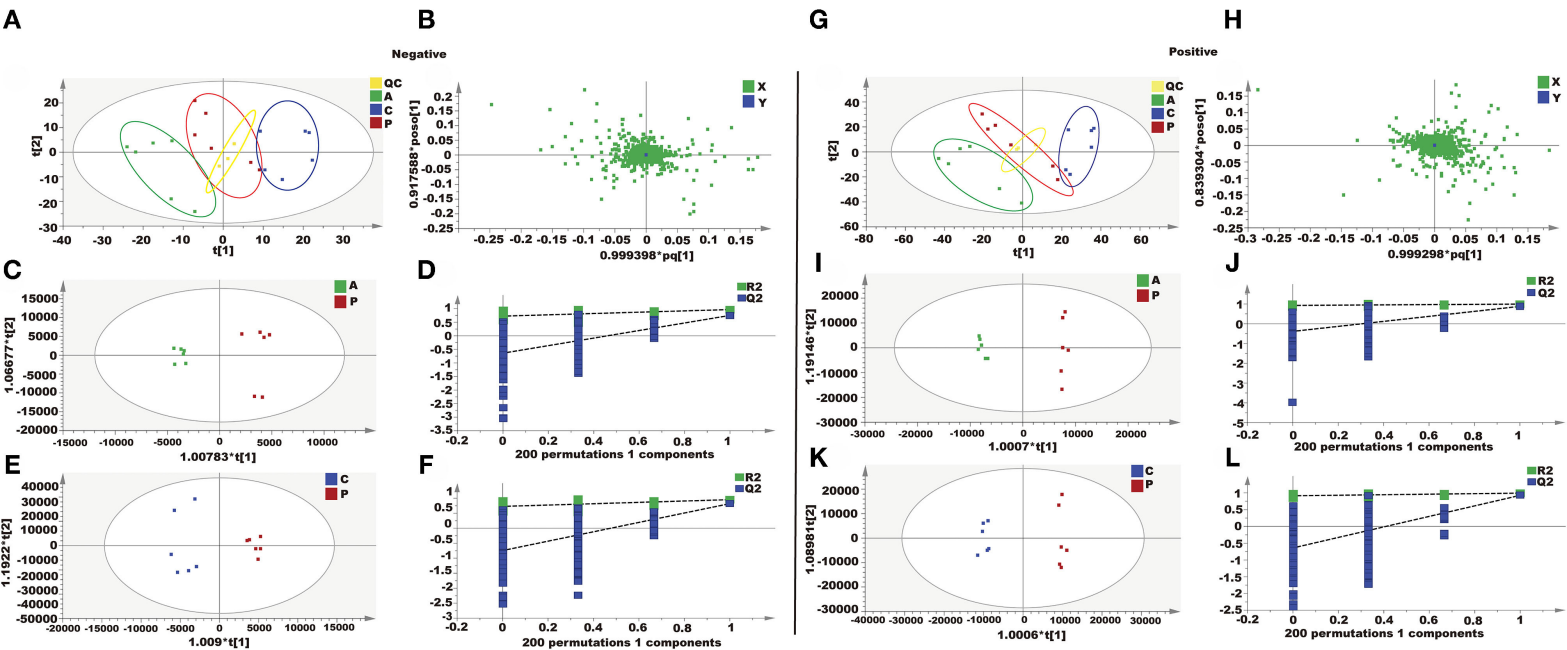
Metabolomics analysis of the effect of AEE on PQ-induced ALI in rats. **(A,G)** PCA score plots based on supernatant of rat liver tissue of the control, PQ and AEE groups in positive and negative modes, ESI+: R^2^ = 0.614, ESI–: R^2^ = 0.589. **(B,H)** The loading plot of AEE and PQ groups in positive and negative modes. **(C,I)** OPLS-DA score plots of the AEE and PQ groups in positive and negative modes, ESI+: R^2^X = 0.566, R^2^Y = 0.994, Q^2^ = 0.878; ESI–: R^2^X = 0.502, R^2^Y = 0.969, Q^2^= 0.752. **(D,J)** Permutation test of the OPLS-DA model, ESI+: the intercepts of R^2^ = 0.926 and Q^2^ = −0.387, ESI–: the intercepts of R^2^ = 0.726 and Q^2^ = −0.640. **(E,K)** OPLS-DA score plots of the control and PQ groups in positive and negative modes, ESI+: R^2^X = 0.592, R^2^Y = 0.993, Q^2^ = 0.927; ESI–: R^2^X = 0.405, R^2^Y = 0.952, Q^2^ = 0.820. **(F,L)** Permutation test of the OPLS-DA model, ESI+: the intercepts of R^2^ = 0.915 and Q^2^ = −0.641, ESI–: the intercepts of R^2^ = 0.727 and Q^2^ = −0.744. Values are presented as the means ± SD where applicable (*n* = 6).

In this study, an unsupervised PCA was performed with the data from three experimental groups. In both positive and negative modes, the first two principal components explained 61.4 and 58.9% of the total variance, respectively. As shown in the PCA plots (Figures 3A,B,G,H), the three groups showed obvious separation in both positive and negative ion modes. In order to further maximize the separation and identification of metabolites, supervised orthogonal partial least squares discriminant analysis (OPLS-DA) was used. Then an OPLS-DA model was established between the PQ group and other groups to enhance the variation. The OPLS-DA score plots presented an obvious separation between the PQ group and other groups without any overlap in either the positive or negative modes (Figures 3C,E,I,K). The R^2^X, R^2^Y, and Q^2^ values of the OPLS-DA model showed that the models were robust and had predictive abilities (Figures 3D,F,J,L).

Differential metabolites contributing to the separation were identified using variable importance in the projection (VIP) value and *p* value. The potential metabolites were screened with a VIP value > l and *p* < 0.05. As shown in Table 1, 32 metabolites were identified as potential metabolites, including dephospho-CoA, taurochenodesoxycholic acid, lysoPC(14:1), chenodeoxyglycocholic acid, PA(22:2), PA(22:2), cholic acid, 5,9,11-trihydroxyprosta-6E,14Z-dien-1-oate, lysoPE(18:2), lysoPE(20:4), lysoPE(16:0), lysoPC(16:0), L-Histidine, pipecolic acid, glycerophosphocholine, acetylglycine, N-(2-Methylpropyl)acetamide, D-Asparagine, hypoxanthine, inosine, xanthosine, L-Phenylalanine, melatonin radical, ophthalmic acid, nonyl isovalerate, glutamylarginine, glutamylleucine, pipecolic acid, S-(PGJ2)-glutathione, L-Octanoylcarnitine, lysoPC(16:0), argininic acid, deoxycholic acid glycine conjugate, N-Undecanoylglycine. After AEE treatment, the levels of these metabolites normalized either due to upregulation or downregulation.

**Table 1:** Statistics of differential metabolites in the rat liver.

**No**	**RT**	**VIP**	**Formula**	**Metabolites**	**SM**	**m/z**	**Fold Change**	
							**PQ/C**	**AEE/PQ**
1	1.036	1.08	C_6_H_9_N_3_O_2_	L-Histidine	ESI+	155.1546	0.86	1.03
2	1.146	2.51	C_6_H_11_NO_2_	Pipecolic acid	ESI+	129.157	0.78	1.16^*^
3	1.154	1.06	C_8_H_20_NO_6_P	Glycerophosphocholine	ESI+	257.223	1.34	0.74^*^
4	1.213	3.14	C_4_H_7_NO_3_	Acetylglycine	ESI+	117.1033	0.77	1.63^*^
5	1.314	1.05	C_6_H_13_NO	N-(2-Methylpropyl)acetamide	ESI+	115.1735	1.04	1.47^*^
6	1.817	2.46	C_4_H_8_N_2_O_3_	D-Asparagine	ESI+	132.1179	0.92	1.55^*^
7	3.638	1.17	C_5_H_4_N_4_O	Hypoxanthine	ESI+	136.1115	1.37	0.92^*^
8	3.646	1.09	C_10_H_12_N_4_O_5_	Inosine	ESI+	268.2261	0.85	1.15^*^
9	4.517	2.84	C_10_H_12_N_4_O_6_	Xanthosine	ESI+	284.2255	0.98	1.85^*^
10	4.627	4.61	C9H11NO2	L-Phenylalanine	ESI+	165.1891	0.46	1.48^*^
11	4.779	2.43	C_13_H_17_N_2_O_3_	Melatonin radical	ESI+	249.2857	0.39	0.75^*^
12	4.959	1.73	C_11_H_19_N_3_O_6_	Ophthalmic acid	ESI+	289.2851	0.40	0.85^*^
13	5.379	1.24	C_14_H_28_O_2_	Nonyl isovalerate	ESI+	228.3709	0.91	0.80
14	5.717	1.42	C_11_H_21_N_5_O_5_	Glutamylarginine	ESI+	303.319	0.49	0.98^*^
15	5.802	1.26	C_11_H_20_N_2_O_5_	Glutamylleucine	ESI+	260.29	0.91	0.64
16	6.368	1.57	C_6_H_11_NO_2_	Pipecolic acid	ESI+	129.157	1.47	1.04^*^
17	6.682	1.07	C_30_H_47_N_3_O_10_S	S-(PGJ2)-glutathione	ESI+	641.773	0.65	1.19^*^
18	9.368	1.04	C_15_H_29_NO_4_	L-Octanoylcarnitine	ESI+	287.3951	0.60	2.18^*^
19	9.419	1.46	C_24_H_50_NO_6_P	LysoPC(P-16:0)	ESI+	479.6307	1.80	3.18^*^
20	10.117	1.06	C_6_H_13_N_3_O_3_	Argininic acid	ESI+	175.1857	0.79	1.78^*^
21	13.354	2.48	C_26_H_43_NO_5_	Deoxycholic acid glycine conjugate	ESI+	449.6233	0.85	0.82
22	13.969	2.42	C_13_H_25_NO_3_	N-Undecanoylglycine	ESI+	243.3425	0.89	1.07
23	4.995	3.96	C_21_H_35_N_7_O_13_P_2_S	Dephospho-CoA	ESI-	687.15	1.95	1.04^*^
24	9.331	5.39	C_26_H_45_NO_6_S	Taurochenodesoxycholic acid	ESI-	499.3	1.44	0.89^*^
25	10.677	1.07	C_22_H_44_NO_7_P	LysoPC(14:1)	ESI-	465.561	2.15	1.04^*^
26	10.776	3.14	C_26_H_43_NO_5_	Chenodeoxyglycocholic acid	ESI-	449.6233	1.97	2.33
27	11.221	2.03	C_47_H_89_O_8_P	PA(22:2)	ESI-	813.195	1.29	1.11
28	12.143	2.50	C_24_H_40_O_5_	Cholic acid	ESI-	408.5714	2.05	0.82^*^
29	14.502	1.11	C_30_H_37_NO_8_	5,9,11-trihydroxyprosta-6E,14Z-dien-1-oate	ESI-	539.625	1.29	0.77^*^
30	14.897	1.61	C_23_H_44_NO_7_P	LysoPE(18:2)	ESI-	477.5717	1.33	0.92^*^
31	14.973	1.15	C_25_H_44_NO_7_P	LysoPE(20:4)	ESI-	501.5931	2.06	0.75^*^
32	15.650	1.69	C_21_H_44_NO_7_P	LysoPE(16:0)	ESI-	453.5503	1.65	1.01^*^

RT, retention time; VIP, variable importance in the projection; SM, scan mode; +, metabolites identified in positive mode; –, metabolites identified in negative mode. Metabolites identified in both positive and negative modes;

* *p < 0.05 compared with the PQ group; C/PQ, control group compared with the PQ group; AEE/PQ, AEE group compared with the PQ group*.

#### Metabolic Pathway Analysis

**Figure 4:**
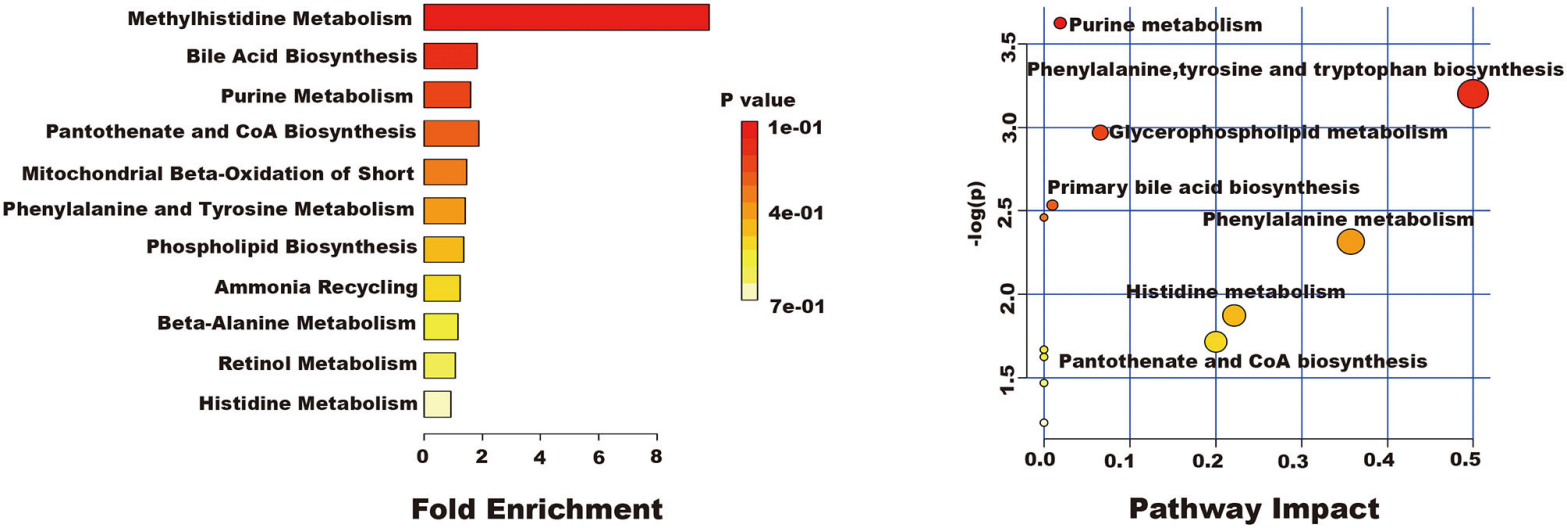
The results of fold enrichment and path analysis of potential metabolites in the supernatant of liver tissue.

The related metabolic pathway analysis was performed on MetaboAnalyst 4.0. The metabolic pathway analysis data are shown as a bar chart and a bubble chart in Figure 4. There are 12 main metabolic pathways: purine metabolism, phenylalanine, tyrosine and tryptophan biosynthesis, glycerophospholipid metabolism, primary bile acid biosynthesis, aminoacyl-tRNA biosynthesis, phenylalanine metabolism, histidine metabolism, pantothenate and CoA biosynthesis, ether lipid metabolism, beta-Alanine metabolism, lysine degradation, cysteine and methionine metabolism. As shown in Figure 4, there are significant differences in metabolic pathways, including Methylhistidine Metabolism, Bile Acid Biosynthesis, Purine Metabolism, Pantothenate and CoA Biosynthesis, Mitochondrial Beta-Oxidation of Short (*p* < 0.05). The influence of the path is mainly concentrated in Phenylalanine, tyrosine and tryptophan biosynthesis, Purine metabolism, Glycerophospholipid metabolism, and Primary bile acid biosynthesis. PQ-induced ALI in rats is mainly reflected in redox reaction and energy metabolism. The results showed that ALI induced by PQ caused metabolic disorder in rats, and AEE could effectively regulate this imbalance.

As shown in Table 1, AEE could increase the levels of L-Histidine, D-Asparagine, and L-Phenylalanine compared with PQ group. Some studies have shown that L-Histidine and D-Asparagine have the effect of anti-apoptosis (31–33). The deficiency of L-Histidine can cause apoptosis through mitochondrial dysfunction, and as a substrate of asparagine biosynthesis, the deficiency of D-asparagine can also promote apoptosis (31, 32). Interestingly, higher concentrations of L-Phenylalanine also inhibited mitochondrial function and cause apoptosis (33). It is necessary to detect the expression of mitochondrial apoptosis-related proteins.

### AEE Decreased the Level of Apoptosis-Related Proteins in Rat Liver Tissue Induced by PQ

**Figure 5:**
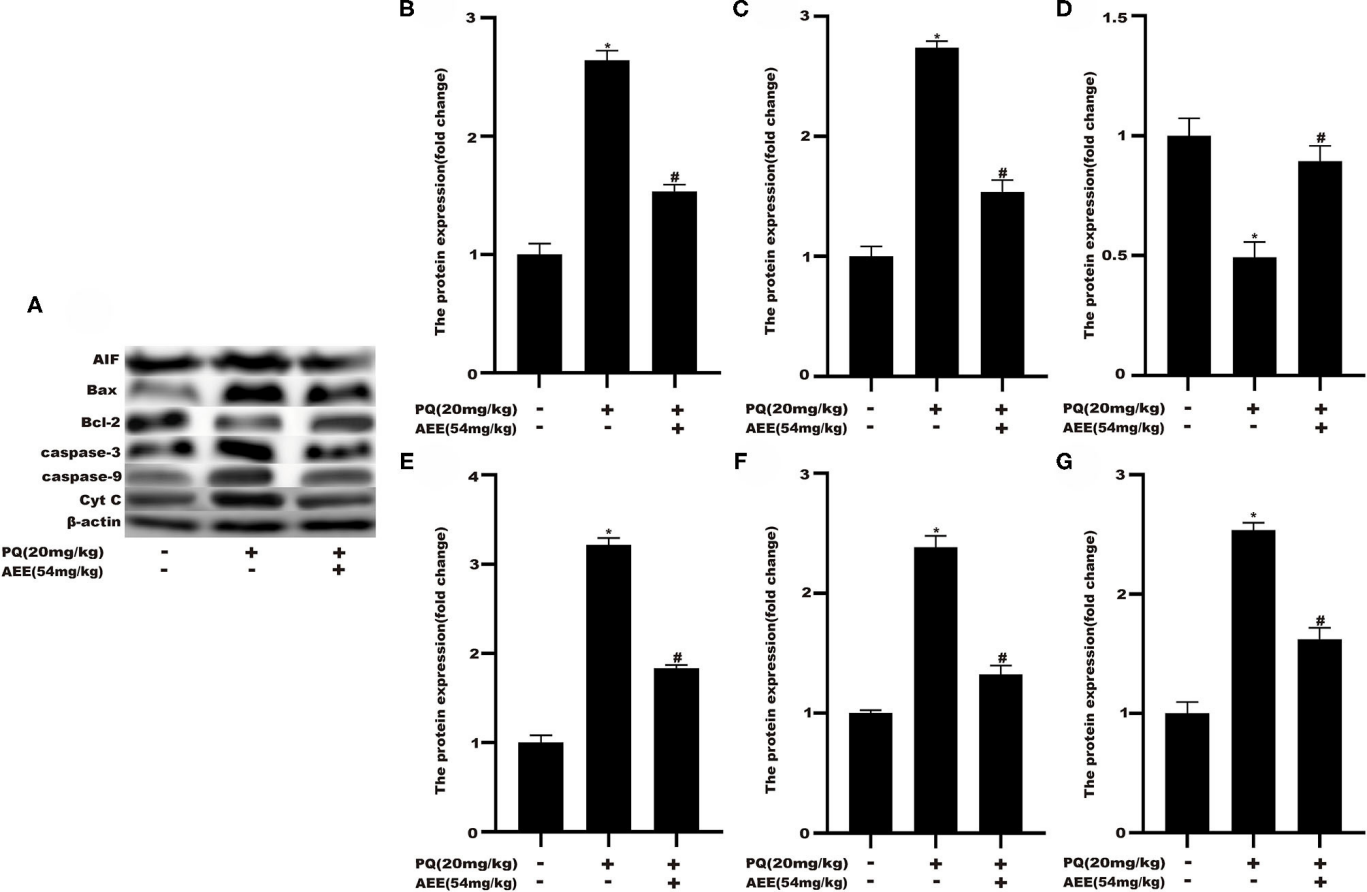
AEE decreased the level of apoptosis-related proteins in rat liver tissue induced by PQ. **(A,B)** The expression of AIF protein in liver tissue of different treatment groups was detected. **(A,C)** The expression of Bax protein in liver tissue of different treatment groups was detected. **(A,D)** The expression of Bcl-2 protein in liver tissue of different treatment groups was detected. **(A,E)** The expression of Caspase-3 protein in liver tissue of different treatment groups was detected. **(A,F)** The expression of Caspase-9 protein in liver tissue of different treatment groups was detected. **(A,G)** The expression of Cyt C protein in liver tissue of different treatment groups was detected. Values are presented as the means ± SD where applicable (*n* = 6). *p < 0.05 compared with the control group; ^#^*p* < 0.05 compared with the PQ group.

To delineate the effector pathways of PQ-induced apoptosis, we examined the expression of mitochondrial apoptosis-related proteins and the expression of caspases, the central executioners of cell apoptosis. Compared with the control group, the expression of Caspase-3, Caspase-9, Bax, Cyt C, and AIF in the model group increased, while the expression of Bcl-2 decreased (Figure 5). In AEE pretreatment group, AEE could inhibit the increase of Caspase-3, Caspase-9, Bax, Cyt C, and AIF induced by PQ, and enhance the expression of Bcl-2. Western blotting analysis showed that AEE reduced the apoptosis of liver cells via inhibiting the expression of apoptosis-related proteins in rat liver tissue induced by PQ.

## Discussion

AEE is synthesized by combining aspirin with eugenol based on the prodrug principal (21). As a new potential compound with anti-inflammatory and antioxidant stress pharmacological activities, AEE plays an active role in many aspects (18–21, 23, 34–38). AEE can prevent tail thrombosis induced by c kappa-carrageenan in rats (19). At the same time, AEE can attenuate thrombus induced with high-fat diet in rats by regulating platelet aggregation, hemorheology, TXB2/6-keto-PGF1α, and blood biochemistry (38). With further study, a rat model of blood stasis was established and it was observed that AEE could alleviate the symptoms of blood stasis in rats (39). It was also found that AEE can inhibit agonist-induced platelet aggregation in rats by regulating PI3K/Akt, MAPK, and Sirt1/CD40L signal pathways (35). AEE has not only the effects of anti-inflammation, anti-thrombosis and anti-blood stasis, but also the effect of anti-atherosclerosis and other cardiovascular diseases. AEE can reduce the oxidative stress of human umbilical vein endothelial cells induced by H_2_O_2_ through mitochondrial-lysosomal axis and Nrf2 signaling pathway, and then reduce the oxidative damage of vascular endothelial cells (18, 23).

PQ poisoning is caused by the selective accumulation of PQ molecules that can cause multiple organ failure and can cause severe damage to the liver (15). Although progress has been made in the comprehensive treatment of PQ poisoning, the mortality rate remains high due to the lack of effective treatment (40, 41). The underlying mechanism of PQ poisoning has not been fully elucidated, but it may be multifactorial. Studies have shown that an important cause of PQ poisoning is the excessive production of ROS (42). The overproduction of reactive oxygen species could cause excessive oxidative stress and oxidant injury in cells (43, 44). ALT and AST are enzymes found in hepatocytes. When the liver cell membrane lipid peroxidation occurs, two enzymes are easily released into the blood. The elevated levels of AST and ALT in liver and serum may indicate PQ-induced ALI. MDA is the end product of lipid peroxidation and its level can be used to assess the extent of damage from peroxidative damage (45–47). Downregulation of AST, ALT, and MDA levels meant that AEE could reduce lipid peroxidation damage. Antioxidant enzymes such as SOD, CAT and GSH-Px play an important role in ROS removal. SOD is the most important antioxidant enzyme for removing H_2_O_2_ from ${\text{O}}_{2}\cdot^{-}$ (48–50). CAT and GSH-Px are the major enzymes that convert H_2_O_2_ to O_2_ and H_2_O (51–54). In the model group, ROS produced by PQ increased MDA levels and decreased SOD, GSH-Px, and CAT levels. After AEE administration, SOD, GSH-Px, and CAT increased. This indicates that AEE could restore ALI in PQ-induced rats via ROS scavenging.

Arginine synthesis and the metabolism of arginine and proline involved in L-arginine may be one of the most important metabolic pathways in which AEE plays a protective role in PQ-induced lung injury. L-arginine is a semi-essential amino acid needed for cell proliferation, and is the substrate of arginase 1 (Arg-1) and inducible nitric oxide synthase (iNOS), which is involved in the oxidative stress of the body to external stimuli. Metabonomic results showed that the biosynthesis pathway of L-arginine was inhibited in PQ group. L-arginine is a scavenger of free radicals in the body (55). L-arginine increases the activity of antioxidant enzymes and reduces the content of MDA by promoting the production of nitric oxide (NO), thus reducing the tissue damage caused by oxidative stress (56). After pretreatment with AEE, the production of L-arginine increased, which in turn promoted the increase of SOD, GSH-Px and CAT. It is suggested that AEE may alleviate PQ-induced lung injury in rats by scavenging excessive ROS.

Glycerophospholipid metabolites, including PC and LysoPE are key components of the lipid bilayer of cells, as well as being involved in metabolism and signaling (57–59). A previous study suggested that various PCs and LysoPEs were significantly increased in rat acute blood stasis model and AEE could significantly inhibit the increase of PC and LysoPE (39). AEE increased high-density lipoprotein cholesterol serum level and decreased low-density lipoprotein cholesterol serum level in hyperlipidemia model induced by high-fat diet. Notably, the elevated TG and TC serum levels were also reversed by AEE. All of the above implied that lipid metabolism was partly restored by AEE.

**Figure 6:**
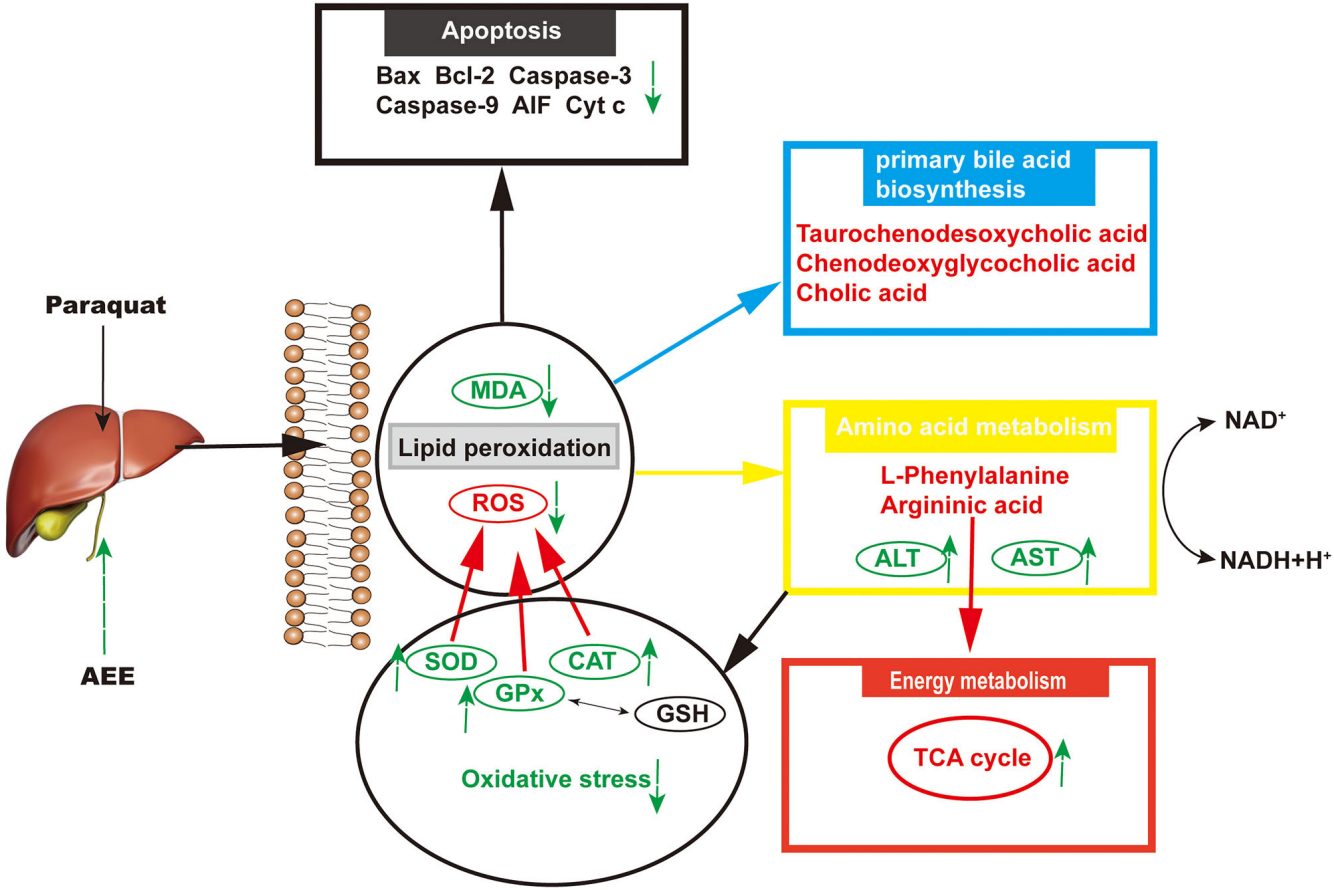
The results of hepatoprotective effect of AEE on ALI rats.

Mitochondrial damage was present due to impaired energy, amino acid, and fatty acid metabolism. The production of ROS can also cause mitochondrial apoptosis (60–62). Therefore, apoptosis may play an important role in the pathogenesis of liver injury. In this study, the hepatic apoptotic cell rate was increased in the model group. The low percentage of hepatic apoptotic cells in the AEE group suggests that AEE enhanced antioxidant activity and attenuated apoptosis. On the other hand, the results of Western blotting analysis suggest that the expression levels of Caspase-9, Bax, Cyt C, Caspase-3, and AIF were decreased, whereas that of Bcl-2 was increased in the AEE group. Figure 6 summarizes the protective effects of AEE on ALI rats. As shown in Figure 6, PQ could induce excessive production of ROS in liver tissue. Excessive ROS could further increase the excessive production of MDA and decrease the activities of antioxidant enzymes such as SOD, CAT, and GSH-Px. The decrease of antioxidant enzyme activity would lead to the release of apoptotic proteins, including Caspase-9, Bax, Cyt C, Caspase-3, and AIF. There is no doubt that when apoptosis occurs, the energy supply of mitochondria in the cell will be insufficient, and the synthesis and metabolism of some amino acids will be hindered. In this study, the metabolism and synthesis of chenodeoxycholic acid, chenodeoxycholic acid, and cholic acid were affected to some extent. Undoubtedly, during the amino acid metabolism process, the levels of L-Phenylalanine and Argininic acid decreased significantly after PQ treatment. The metabolism of amino acids would further affect the energy metabolism of cells, especially in the TCA cycle.

## Conclusion

AEE exhibited protective effects on PQ-induced ALI. The underlying mechanism was correlated with antioxidants that regulate amino acid, phospholipid and energy metabolism metabolic pathway disorders and alleviate liver mitochondria apoptosis.

## Data Availability Statement

The original contributions presented in the study are included in the article/supplementary materials, further inquiries can be directed to the corresponding author/s.

## Ethics Statement

The animal study was reviewed and approved by the Institutional Animal Care and Use Committee of Lanzhou Institute of Husbandry and Pharmaceutical Science of Chinese Academy of Agricultural Sciences (Approval No. NKMYD201907018; Approval Date: 18 July 2019).

## Author Contributions

Z-DZ designed and performed the experiments. Y-JY synthesized and purified AEE. X-WL, S-HL, and ZQ assisted with the animal experiments. J-YL supervised the study and revised the manuscript.

## Conflict of Interest

The authors declare that the research was conducted in the absence of any commercial or financial relationships that could be construed as a potential conflict of interest.
